# Insights from Egyptian ticagrelor study in patients who presented with acute coronary syndrome (ETS in ACS)

**DOI:** 10.1186/s43044-022-00290-w

**Published:** 2022-06-21

**Authors:** Hesham S. Taha, Hossam Kandil, Nabil Farag, Amr Zaki, Hossam Mahrous, Mirna M. Shaker

**Affiliations:** 1 Department of Cardiology, Faculty of Medicine, Cairo University, 27 Nafezet Sheem El Shafae St Kasr Al Ainy, Cairo, 11562 Egypt; 2 Ain-Shams University, Cairo, Egypt; 3 Alexandria University, Alexandria, Egypt

**Keywords:** Antiplatelet therapy, Acute coronary syndrome, Cardiovascular death, Bleeding

## Abstract

**Background:**

Dual antiplatelet therapy with aspirin and a thienopyridine is used to prevent thrombotic complications of acute coronary syndrome (ACS) and percutaneous coronary interventions (PCI). Ticagrelor is an oral, reversible inhibitor of the adenosine diphosphate receptor P2Y12 with a faster onset and more potent platelet inhibition than clopidogrel. A study was needed to evaluate the efficacy and safety of generic ticagrelor in Egyptian patients.

**Results:**

This multicenter study included 830 patients aged above 40 years and diagnosed with ACS, with or without ST segment elevation during the preceding 6 months. They received generic ticagrelor (Thrombolinta, Global Napi Pharmaceutical Company, Egypt) (180 mg loading dose, 90 mg twice daily thereafter), added to aspirin 75–100 mg daily. The mean age of our study population was 57.5 (8.3) years and 38.3% were females. Hypertension, diabetes mellitus, dyslipidemia and previous coronary revascularization were present in 70.7%, 59.2%, 80.7% and 31% of the patients, respectively, and 42.5% were current smokers. The qualifying event was unstable angina, non-ST segment elevation myocardial infarction and ST segment elevation myocardial infarction in 54%, 21.8% and 24.2% of the patients, respectively. At 6 months, the primary efficacy end point—a composite of cardiovascular death, myocardial infarction and stroke—occurred in 3.4% of patients, while the secondary efficacy endpoint—a composite of the primary efficacy endpoints with the addition of hospitalization for unstable angina and urgent revascularization—occurred in 15.3%. Cardiovascular death occurred in 1.2% of the patients, myocardial infarction in 0.8%, stroke in 1.3%, hospitalization for UA in 8.1% and urgent revascularization in 3.9%. TIMI major bleeding occurred in 1.2% of patients, intracranial hemorrhage in 0.2% and TIMI minor bleeding in 13.3%. No significant difference was found between patients who underwent PCI at baseline and those who were treated conservatively regarding the primary (14 patients in each group, *P* = 0.931) and secondary (62 vs. 65 patients, *P* = 0.946) efficacy endpoints.

**Conclusions:**

In patients who had an ACS during the 6 months preceding enrollment, treatment with generic ticagrelor led to a low rate of cardiovascular death, myocardial infarction and stroke with a minor increase in the risk of major bleeding.

## Background

Activated platelets have a key role in the pathology of cardiovascular (CV) ischemic events [1]. Aspirin reduces the risk of ischemic events among patients who present with an acute coronary syndrome (ACS). The addition of a P2Y12 receptor antagonist to aspirin has been shown to reduce further the risk of ischemic events in this population in the first year after an ACS [2].

Ticagrelor is an oral, reversible, direct-acting, inhibitor of the adenosine diphosphate (ADP) receptor P2Y12, which has a more rapid onset and a more potent platelet inhibition than clopidogrel. When added to aspirin for 1 year after an ACS, ticagrelor, at a dose of 90 mg twice daily, reduced the rate of major adverse CV events including CV death, as compared with clopidogrel at a dose of 75 mg once daily [3]. Current guidelines recommend adding a P2Y12 receptor antagonist to aspirin for the first year after an ACS [4].

In low- and middle-income countries, medication price is commonly an issue and is an important cause of lack of compliance to long-term treatment; hence the importance of low-cost generic medications provided they are effective and safe.

A study was needed to evaluate the efficacy and safety of generic ticagrelor in Egyptian patients in a real-world setting.

## Methods

### Study design and oversight

The protocol was designed to test the hypothesis that the addition of generic ticagrelor to aspirin in Egyptian patients with a history of ACS would reduce ischemic events. All data were reported using a case report format and collected data were transferred to an excel sheet. The study steering committee overviewed reported data including all information corresponding to the clinical endpoints and serious adverse events.

### Study population

Patients were included in the study if they were hospitalized for an ACS, with or without ST segment elevation, during the 6 months prior to enrollment, with or without coronary intervention. They were at least 40 years old and tolerant to acetylsalicylic acid (aspirin) at a dose of 75–100 mg/d. Eligible patients had to sign an informed consent to participate in the study.

Patients were excluded if there was a planned use of another P2Y12 receptor antagonist, dipyridamole, cilostazol or anticoagulant therapy during the study period. They were also not considered for inclusion if they had a bleeding disorder, a history of an ischemic stroke, an intracranial bleeding, a central nervous system tumor, an intracranial vascular abnormality or any other contraindication to the use of ticagrelor. They were also considered ineligible if they had had a gastrointestinal bleeding within the preceding 6 months or major surgery within the preceding 30 days and if they were planned to undergo elective major non-CV procedures within the following 6 months. Other exclusion criteria included severe liver disease, end-stage kidney disease on dialysis, being at risk of bradycardia and females in the childbearing period who were not using birth control methods or who were pregnant, lactating or planning for pregnancy.

At the time of screening a patient might or might not have been receiving a P2Y12 receptor antagonist. Patients who were on a P2Y12 receptor antagonist other than the study medication were shifted to the study drug ticagrelor at study entry.

#### Study treatment

All patients included in the study received ticagrelor (Thrombolinta, Global Napi Pharmaceutical Company) orally at a dose of 90 mg twice daily. Patients received a loading dose of Ticagrelor (180 mg) at initiation of treatment as per guideline recommendations [7], and at the discretion of the treating physician, depending on whether the patient was antiplatelet naïve or not, the timing from ACS index event and if he/she was undergoing PCI or not. All patients received aspirin at a dose of 75–100 mg daily.

### End points

The primary efficacy end point was the composite of CV death, myocardial infarction or stroke. Secondary efficacy end points included the individual components of the primary end points and the additional end points of urgent coronary revascularization, hospitalization for unstable angina (UA) and transient ischemic attack (TIA). CV death was defined as sudden cardiac death due to a CV cause or death for which there was no clearly documented non-CV cause (presumed CV death). Myocardial Infarction was defined using the Fourth Universal Definition of MI Expert Consensus Document [5] with detection of a rise and/or fall of cardiac troponin (cTn) with at least one value above the ninety-ninth percentile and with at least one of the following: symptoms of acute myocardial ischemia, new ischemic electrocardiographic (ECG) changes, development of pathological *Q* waves, imaging evidence of new loss of viable myocardium or new regional wall motion abnormality in a pattern consistent with an ischemic etiology, or identification of a coronary thrombus by angiography including intracoronary imaging or by autopsy (not for types 2 and 3). Stroke was defined as an acute episode of neurologic dysfunction attributed to a central nervous system vascular cause and documented by imaging. TIA was defined as a transient episode of neurological dysfunction caused by focal brain, spinal cord or retinal ischemia, without acute infarction.

The primary safety end point was TIMI major bleeding. Other safety end points included intracranial hemorrhage and minor bleeding and other side effects. TIMI major bleeding was defined as intracranial bleeding, clinically overt signs of hemorrhage associated with a drop in hemoglobin (Hgb) of ≥ 5 g/dL (or, when hemoglobin is not available, a fall in hematocrit of ≥ 15%) or fatal bleeding (a bleeding event that directly led to death within 7 days). Minor bleeding was defined as any overt bleeding or hemorrhagic event or drop in hemoglobin that does not meet the criteria above [6]. The steering committee oversaw the efficacy and safety end- points.

### Follow-up

In addition to the baseline recruitment visit (visit 0), outpatient visits were scheduled at 3 and 6 months. The study was designed to continue for 6 months, however dual antiplatelet treatment (DAPT) regimen was prescribed, as per guidelines, for at least 12 months from the index ACS event except if there was a contraindication to the continuation of the DAPT regimen. If a patient was not able to come for the follow-up visit, communication could be through telephone/mobile.

### Enrollment sites

To cover diverse populations from different parts of the nation, patients were recruited from seven different governorates. Each patient was given a code; the first figure represented the governorate, the second was specific for the recruiting physician and the third for the patient.

### Statistical analysis

Continuous variables were expressed as mean ± SD and discrete variables as absolute values and percentages. Analyses were performed using SPSS software V.23 (IBM, Armonk, New York, USA).

The primary efficacy variable was the time to the first occurrence of the composite of death from vascular causes, myocardial infarction, stroke or hospitalization for ACS.

Safety analyses included all the patients who underwent randomization and received at least one dose of study drug. The primary safety end point was the first occurrence of any major bleeding event. Additional safety end points included minor bleeding, dyspnea, bradyarrhythmia and any other clinical adverse events.

## Results

Overall, 830 patients from seven Egyptian governorates were recruited, during the period from April to August 2020. The follow-up period ended in March 2021. The mean time from the index ACS event to enrollment was 2.1 (± 1.57) months, with 45.8% of the patients enrolled from the outset, and of those 53.8% underwent PCI at baseline. Five patients did not attend any of the follow-up visits, two patients did not attend the first follow-up visit and 23 patients did not attend the second visit. Baseline characteristics of all included patients are shown in (Table 1). The qualifying event for inclusion was unstable angina (UA) in 54%, non-ST elevation myocardial infarction (NSEMI) in 21.8% and ST elevation myocardial infarction (STEMI) in 24.2%. The overall rate of adherence to the study drug was 98.1% at visit one and 94.5% at the end of the follow-up period.

**Table 1:** Baseline characteristics of all included patients

	Patients
Number	830
Age, mean (years) (SD)	57.5 (8.3)
Females *n* (%)	318 (38.3)
Smoking *n* (%)	353 (42.5)
Hypertension	587 (70.7)
Diabetes mellitus	491 (59.2)
Dyslipidemia	670 (80.7)
Previous PCI or CABG	257 (31)
Prior CVA	132 (15.9)
*Medications at baseline*	
Aspirin *n* (%)	758 (91.3)
PT2Y 12 inhibitors *n* (%)	739 (89)
Statins *n* (%)	705 (84.9)
Beta-blockers *n* (%)	672 (81)
ACEI/ARB/ARNI *n* (%)	540 (65.1)
PPI *n* (%)	514 (61.9)
*Coronary interventions at baseline*	
Coronary angiography *n* (%)	513 (61.8)
PCI *n* (%)	401 (48.3)
PCI to single vessel *n* (%)	278 (33.5)
PCI to multi-vessel *n* (%)	106 (12.8)
PCI to LMT *n* (%)	12 (1.4)

*CABG* Coronary artery bypass graft; *CVA* Cerebrovascular accident; *LMT* Left main trunk; *n* Number; *NSTEMI* Non-ST elevation myocardial infarction; *PCI* Percutaneous coronary intervention; *PPI* Proton pump inhibitor; *SD* Standard deviation; *STEM* ST elevation myocardial infarction; *UA* Unstable angina

### Efficacy

**Fig. 1:**
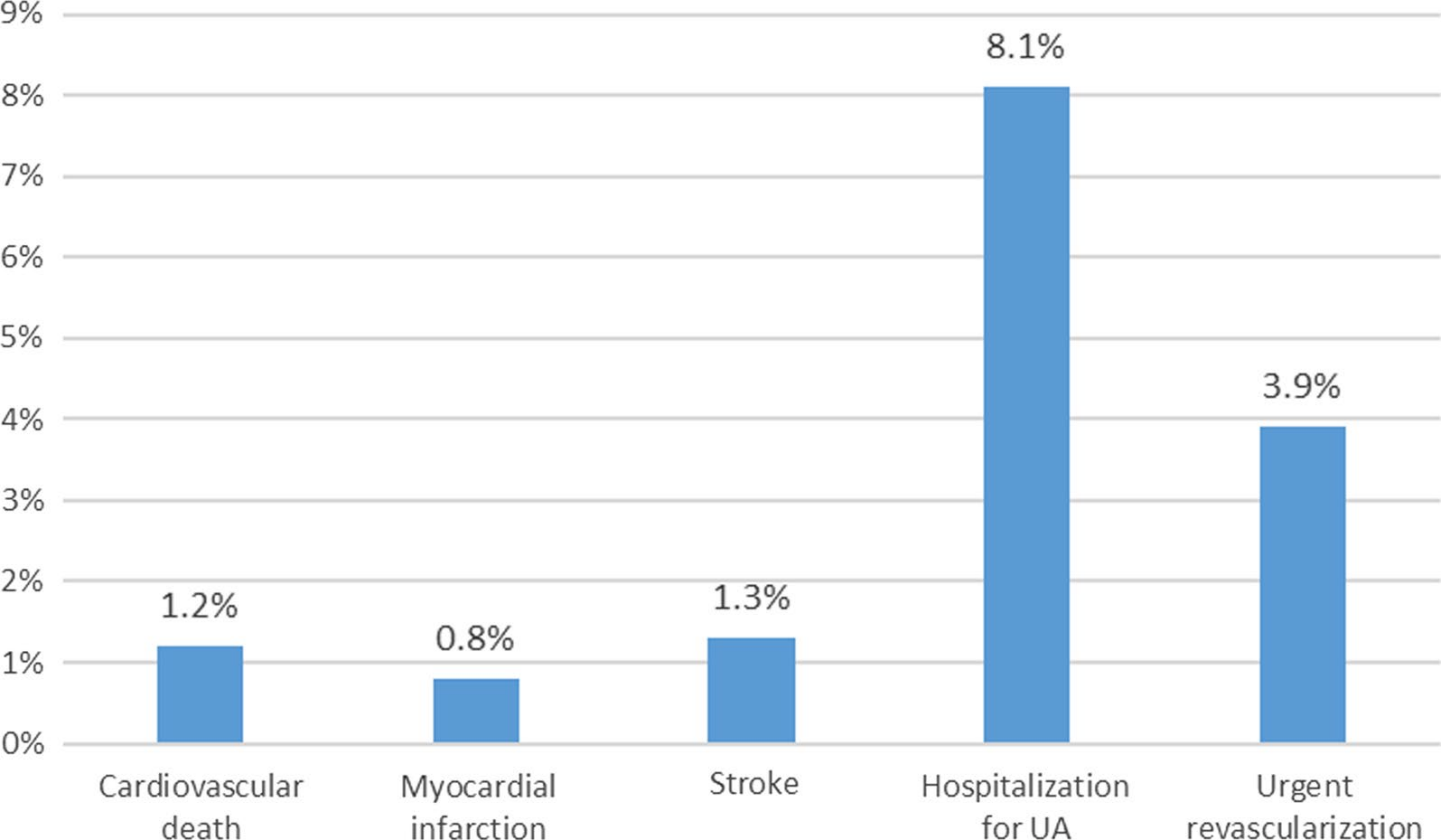
Incidence of primary and secondary efficacy endpoints in all included patients

The primary efficacy end points (composite of CV death, myocardial infarction and stroke) occurred in 3.4% (*n* = 28) of the patients at 6 months. Only seven events occurred in the first 3 months and the remaining 21 events occurred in the last 3 months of the follow-up period. The secondary efficacy endpoints, including the composite of the primary efficacy endpoints with the addition of hospitalization for UA and urgent revascularization, occurred in 15.3% (*n* = 127) of the patients, CV death occurred in 1.2% (*n* = 10), myocardial infarction in 0.8% (*n* = 7), stroke in 1.3% (*n* = 11), hospitalization for UA in 8.1% (*n* = 67) and urgent revascularization in 3.9% (*n* = 32) of the patients (Fig. 1).

### Safety

**Fig. 2:**
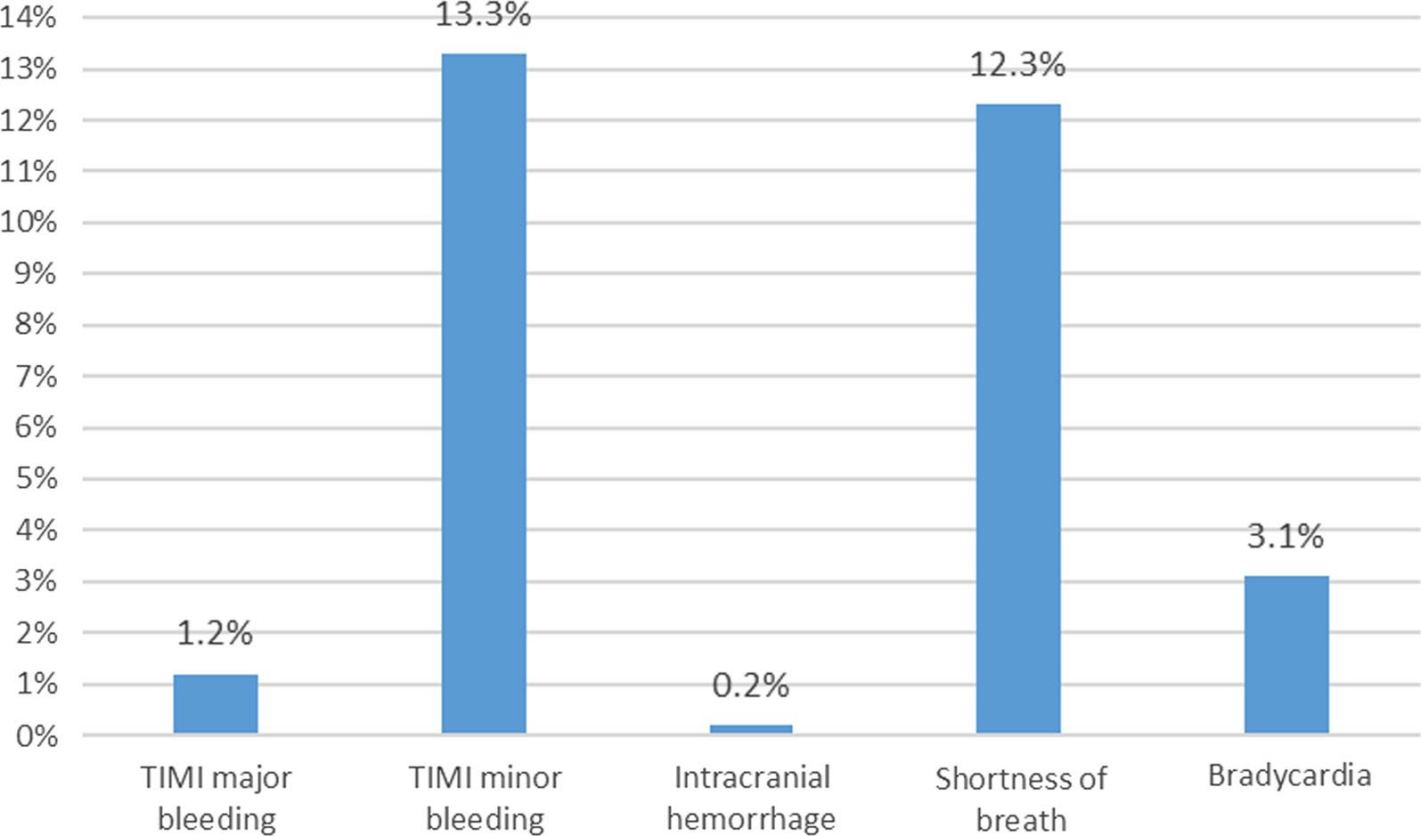
Incidence of safety endpoints in all included patients

The primary safety endpoint (TIMI major bleeding) occurred in 1.2% (*n* = 10) of the patients. Regarding the other safety endpoints, intracranial hemorrhage occurred in 0.2% (*n* = 2), TIMI minor bleeding in 13.3% (*n* = 110), shortness of breath in 12.3% (*n* = 102) and bradycardia in 3.1% (*n* = 26) of the patients (Fig. 2).

Comparing patients who underwent PCI at baseline with those who were treated conservatively, there was no statistically significant difference between the two groups regarding the primary (*P* = 0.931) or major secondary (*P* = 0.946) efficacy endpoints, however urgent revascularization was higher in the PCI group (*P* = 0.027), as well as complications including TIMI major bleeding (*P* = 0.009) and TIMI minor bleeding (*P* = 0.002) (Table 2).

**Table 2:** Efficacy and safety outcomes in patients who underwent PCI at baseline with those who did not undergo PCI

	PCI	No PCI	*P* value
Number (%)	401 (48.3)	429 (51.7)	
Composite primary endpoints (*n*)	14	14	0.931
Secondary efficacy endpoints (*n*)	62	65	0.946
Cardiovascular death (*n*)	4	6	0.348
Myocardial infarction (*n*)	5	2	0.234
Stroke (*n*)	7	4	0.328
Hospitalization for UA (*n*)	33	34	0.879
Urgent revascularization (*n*)	22	10	0.027
TIMI major bleeding (*n*)	9	1	0.009
TIMI minor bleeding (*n*)	71	39	0.002
Intracranial hemorrhage (*n*)	2	0	0.149
Dyspnea (*n*)	64	38	0.004
Bradycardia (*n*)	20	6	0.002

Multiple logistic regression analyses were performed to detect factors predicting the occurrence of the composite primary endpoints (CV death, myocardial infarction and stroke). Among many factors [age, gender, diabetes mellitus, dyslipidemia, hypertension, previous cerebrovascular accident (CVA), previous coronary revascularization, smoking, type of qualifying event and PCI at baseline] included in the analysis, only previous CVA and previous PCI/coronary artery bypass graft (CABG) significantly predicted the occurrence of the composite primary endpoints by 3.2 (95% CI 1.04–9.96, *P* = 0.041) and 3.3-folds (95% CI 1.09–10.12, *P* = 0.035) , respectively.

Mean time of enrollment after ACS was 2.1 months (1.57 SD), with 45.8% of the patients enrolled during index hospitalization for the qualifying event, and of those 53.8% underwent PCI at baseline. Comparing patients who were enrolled from the onset of ACS (in the first month after ACS) with those enrolled later (from second to sixth month), there was no statistically significant difference between the two groups regarding the primary (2.5% vs. 1.8%, *P* = 0.548) or secondary (10.8% vs. 7.6% patients, *P* = 0.168) efficacy endpoints. Moreover, there was no statistically significant difference between the two groups regarding TIMI major bleeding (1.1% vs. 1.5%, *P* = 0.641) or TIMI minor bleeding (13.7% vs. 11.5% patients, *P* = 0.406) (Table 3).

**Table 3:** Efficacy and safety outcomes in patients who were enrolled in the first month after ACS compared to those who were enrolled from the second to the sixth months after ACS

	First month	Second to sixth months	*P* value
Percentage	45.8	54.2	
Composite primary endpoints (%)	2.5	1.8	0.548
Secondary efficacy endpoints (%)	10.8	7.6	0.168
Cardiovascular death (%)	0.7	0.9	0.802
Myocardial infarction (%)	0.7	1.5	0.360
Stroke (%)	2.5	0.6	0.051
Hospitalization for UA (%)	7.9	6.4	0.458
Urgent revascularization (%)	4.3	3.9	0.786
TIMI major bleeding (%)	1.1	1.5	0.641
TIMI minor bleeding (%)	13.7	11.5	0.406
Intracranial hemorrhage (%)	0.4	0.3	0.899
Dyspnea (%)	11.5	9.4	0.394
Bradycardia (%)	4	3.6	0.831

## Discussion

We conducted the “Egyptian Ticagrelor Study in patients who presented with Acute Coronary Syndrome (ETS in ACS)” on 830 patients from seven Egyptian governorates to determine the efficacy and safety of generic ticagrelor in patients who developed ACS during the 6 months prior to enrollment.

The primary end point (composite of CV death, myocardial infarction and stroke) occurred in 3.37% of patients at 6 months. The mean time to enrollment in our study was 2.1 (+ 1.57) months, with 45.8% of the patients enrolled during index hospitalization for the qualifying event. This is in contrast to the PLATO trial in which the primary endpoint occurred in 9.8% of patients in the ticagrelor group compared to 11.7% in those receiving clopidogrel. However, there were some differences between the two trials; in the latter all patients were included within 24 h of the diagnosis of ACS, the follow-up was for one year and the median age of the patients was higher [3].

In our study, the occurrence of the primary endpoint events was unevenly distributed during the follow-up period with 0.84% occurring in the first 3 months and 2.53% occurring in the second 3 months of the follow-up period. In the PLATO trial, death from vascular causes, MI and stroke in days 1–30 was 4.8%, with slightly more event rate during days 31–360 at 5.3% [3].

The secondary efficacy endpoints were relatively low in frequency in our study; the composite of the primary efficacy endpoints with the addition of hospitalization for unstable angina and urgent revascularization occurred in 15.3% of patients. CV death occurred in 1.2% in our 6-month study, compared to vascular death of 4.8% over one year in the PLATO trial, myocardial infarction in 0.8% compared to 5.8% in the PLATO trial. Hospitalization for unstable angina in our study occurred in 8.1%, while urgent revascularization occurred in 3.9% of patients. In the PLATO trial recurrent coronary ischemia occurred in 5.8% [3].

The rate of stroke was also relatively low during the study period (1.3% at 6 months of follow-up) compared to 1.5% during the one year PLATO study in the Ticagrelor arm [3].

The primary safety endpoint (TIMI major bleeding) occurred in 1.2% (*n* = 10) of our patients. Other safety endpoints including intracranial hemorrhage occurred in 0.2%. However, in the one year PLATO trial TIMI major bleeding occurred in 7.9% of the patients in the ticagrelor arm and 7.7% of the patients in the clopidogrel arm [3].On the other hand, in the PEGASUS TIMI 54 trial, the rates of fatal bleeding or non-fatal intracranial hemorrhage were less than 1% over a 3-year period in all three groups in this trial. The study, however, addressed a different population type and excluded patients who were at high bleeding risk [7].

As regards dyspnea, it occurred in 12.3% of our study population, which is in concordance with most of the published literature on dyspnea incidence in patients treated with ticagrelor [3, 8, 9]. Yet, most episodes were short-lived and discontinuation of the study drug was not reported in any of our patients. To the contrary, in the PEGASUS TIMI 54 trial, both the 60 mg and 90 mg ticagrelor doses also caused dyspnea, but the discontinuation rates of the study drug were significantly higher, as compared with placebo. The fact that the patients were recruited 1–3 years following the index event may have contributed to a higher discontinuation rate of the drug on the occurrence of any side effect.

Concerning bradycardia, it was reported in 3.1% of the study population with no syncopal events or need for pacemaker implantation. Seventy-seven percent of them were on beta-blockers which might have exaggerated the bradycardia effects of ticagrelor. In the PLATO trial, bradycardia occurred in 4.4% of the ticagrelor treated patients and 1.1% developed syncope. Holter monitoring detected more ventricular pauses during the first week in the ticagrelor group than in the clopidogrel group, but such episodes were infrequent at 30 days and were rarely associated with symptoms [3].

Comparing patients who underwent PCI at baseline and those who were treated conservatively, there was no statistically significant difference between the two groups regarding the primary or secondary efficacy endpoints. Similarly, in the PLATO trial, the benefits of ticagrelor were seen regardless of whether an invasive or a noninvasive management was planned [3].

Multiple logistic regression analyses showed that only previous CVA and previous PCI/CABG significantly predicted the occurrence of the composite primary endpoints, emphasizing the fact that higher risk patients are at a higher risk of developing complications.

In contrast to the PLATO trial, in which all patients were enrolled within 24 h from onset of ACS, the mean time of enrollment after ACS in our study was 2.1 months (1.57 SD) with 45.8% of the patients enrolled during index hospitalization for the qualifying event. However, there was no statistically significant difference between those enrolled from the onset of ACS with those who were enrolled later (from the second to the sixth month) for both the primary efficacy endpoints and safety endpoints. In the PLATO trial the treatment effects were the same in the short term (days 0–30) and in the longer term (days 31–360).

In our study, the overall rate of adherence to the study drug was 98.1% at visit one and 94.5% at the end of the follow-up period. In PLATO, premature discontinuation of the study drug was relatively high and occurred in 23.4% of patients in the ticagrelor group over one year [3].

In the PLATO trial, there was a difference in results between patients enrolled in North America and those enrolled elsewhere [3], and although no clear explanation was found, this put forward the question whether geographic differences or practice patterns influenced the effects of the randomized treatments. It also raised the importance of conducting trials on ticagrelor in our region where data on its efficacy and safety are quite scarce.

This study has the limitations of being a non-randomized trial, and lacking a comparative arm which could have better been brand ticagrelor. Nevertheless, the study gave us important insights on the type of ACS patients we may be seeing in our region, as well as a chance to compare our results with those reported in the literature. Another limitation was the intermediate term duration of the follow-up which was only 6 months and that it was allowed to enroll patients from day one of the diagnosis of ACS up to 6 months from index event. However, the design and duration of the study were preset in the protocol. The study included patients who recently underwent PCI and some of the investigators could have refrained from giving these patients a generic P2Y12 inhibitor newly introduced to the market, although it was approved by the national authorities to be marketed for such an indication. Also, as per protocol, any ACS patient whether he/she underwent PCI or not, would be eligible for 12 months of DAPT, so if a patient would be recruited at 6 months post-ACS he should continue for at least another 6 months (study duration) and still be within the guideline recommended DAPT duration.

## Conclusions

In patients who had an ACS with or without ST segment elevation, whether they underwent coronary intervention or not, treatment with generic ticagrelor was effective and safe.

## Data Availability

The data set supporting the results and conclusions of this article will be available from the corresponding author on request.

## Abbreviations

ACS: Acute coronary syndrome
ADP: Adenosine diphosphate
cTn: Cardiac troponin
CABG: Coronary artery bypass graft
CV: Cardiovascular
CVA: Cerebrovascular accident
DAPT: Dual antiplatelet treatment
ECG: Electrocardiography
LMT: Left main trunk
*N*: Number
NSTEMI: Non-ST segment elevation myocardial infarction
PCI: Percutaneous coronary interventions
PPI: Proton pump inhibitor
STEMI: ST segment elevation myocardial infarction
SD: Standard deviation
TIMI: Thrombolysis in myocardial infarction
TIA: Transient ischemic attack
UA: Unstable angina
